# Impact of admission serum ionized calcium levels on risk of acute kidney injury in hospitalized patients

**DOI:** 10.1038/s41598-020-69405-0

**Published:** 2020-07-23

**Authors:** Charat Thongprayoon, Wisit Cheungpasitporn, Api Chewcharat, Michael A. Mao, Tarun Bathini, Saraschandra Vallabhajosyula, Sorkko Thirunavukkarasu, Kianoush B. Kashani

**Affiliations:** 1 Division of Nephrology and Hypertension, Department of Medicine, Mayo Clinic, 200 First St SW, Rochester, MN 55905 USA; 2 Division of Nephrology, Department of Internal Medicine, University of Mississippi Medical Center, Jackson, MS USA; 3 Division of Nephrology and Hypertension, Mayo Clinic, Jacksonville, FL USA; 4 Department of Internal Medicine, University of Arizona, Tuscon, AZ USA; 5 Department of Cardiovascular Medicine, Mayo Clinic, Rochester, MN USA; 6 Division of Pulmonary and Critical Care Medicine, Department of Medicine, Mayo Clinic, Rochester, MN USA

**Keywords:** Nephrology, Kidney, Kidney diseases

## Abstract

This study aimed to investigate the risk of acute kidney injury (AKI) in hospitalized patients based on admission serum ionized calcium levels. This is a cohort study of all hospitalized adult patients, from January 2009 to December 2013 at a tertiary referral hospital, who had available serum ionized calcium at the time of admission. We excluded patients who had end-stage kidney disease or AKI at admission. We stratified admission serum ionized calcium into 6 groups; ≤ 4.39, 4.40–4.59, 4.60–4.79, 4.80–4.99, 5.00–5.19, and ≥ 5.20 mg/dL. We used serum creatinine criterion of KDIGO definition for diagnosis of AKI. We performed logistic regression analysis to assess the risk of in-hospital AKI occurrence based on admission serum ionized calcium, using serum ionized calcium of 5.00–5.19 mg/dL as the reference group. We studied a total of 25,844 hospitalized patients. Of these, 3,294 (12.7%) developed AKI in hospital, and 622 (2.4%) had AKI stage 2 or 3. We observed a U-shaped association between admission serum ionized calcium and in-hospital AKI, with nadir in-hospital AKI was in serum ionized calcium of 5.00–5.19 mg/dL. After adjustment for confounders, low serum ionized calcium of 4.40–4.59, ≤ 4.39 mg/dL and elevated serum ionized calcium ≥ 5.20 mg/dL were associated with increased risk of AKI with odds ratio of 1.33 (95% CI 1.14–1.56), 1.45 (95% CI 1.21–1.74), and 1.26 (95% CI 1.04–1.54), respectively. Both hypocalcemia, and hypercalcemia at the time of admission were associated with an increased risk of hospital-acquired AKI.

## Introduction

Acute kidney injury (AKI) is a common complication among hospitalized patients with approximate incidence of 20%^1–4^. Importantly, AKI is independently associated with in-hospital mortality and de novo or progression of chronic kidney disease^2,3,5,6^. Early detection and management for AKI and its complications are needed to decrease its dire clinical impacts. Calcium homeostasis is commonly disturbed as a consequence of AKI^7^. However, whether calcium, specifically ionized calcium plays a role in the development of AKI is unclear.

Ionized calcium is an essential mineral required for many physiologic functions in the body, from cellular function, intracellular messenger transduction, hormonal activity, to cardiac function and neuronal activity^8–11^. From the kidney standpoint, AKI in the setting of hypercalcemia can arise via several mechanisms such as volume depletion from polyuria (decreased collecting duct cells response to vasopressin) and direct alterations of intravascular tone^12–14^. Besides, persistent hypercalcemia can lead to calcium deposits in the kidneys or nephrocalcinosis^15^. Previous studies have shown associations between electrolyte disturbances and a higher incidence of AKI among hospitalized patients, specifically an imbalance in total calcium, phosphate, and calcium-phosphorus product^16,17^. These abnormalities seem to directly or indirectly correlate with serum ionized calcium^18^. Therefore, serum ionized calcium might be a useful marker to predict AKI and might also be independently associated with AKI. However, data on the impact of serum ionized calcium on incidence of AKI among hospitalized patients is limited.

Our study aimed to assess the association between admission serum ionized calcium and risk of in-hospital AKI among hospitalized patients retrieving data from an electronic medical record system.

## Materials and methods

### Study population

This historical cohort study, conducted at Mayo Clinic, Rochester, MN, USA, included all hospitalized adult patients from January 1st, 2009 to December 31st, 2013 who had available serum ionized calcium within 24 hours of hospital admission. We excluded individuals with end-stage kidney disease (ESKD), patients whose AKI was present at the time of admission, and those whose serum creatinine was not available during hospitalization. For patients with recurrent admissions, only the first hospital admission during the study period was analyzed. Mayo Clinic Institutional Review Board reviewed and approved this project (IRB number 15-000024) and waived informed consent due to the minimal risk nature of this study. The study was conducted in accordance with the relevant guidelines and regulations.

### Data collection

Institutional electronic medical record system was used to automated retrieval of data collection for clinical characteristics, demographic information, and laboratory data. The predictor of interest was the admission serum ionized calcium, defined as the first serum ionized calcium measured within 24 hours of hospital admission. Estimated glomerular filtration rate (eGFR) was calculated based on age, sex, race, and baseline serum creatinine, using the Chronic Kidney Disease Epidemiology Collaboration (CKD-EPI) equation^19^. The Charlson Comorbidity Index was calculated to assess comorbidity burden at the time of admission^20^. Principal diagnoses were grouped based on admission ICD-9 codes. ESKD was identified using ICD-9 diagnosis code or eGFR of ≤ 15 mL/min/1.73 m^2^.

### Clinical outcomes

The outcome of interest was the occurrence of acute kidney injury (AKI) within 7 days of hospital admission. AKI was diagnosed by the KDIGO serum creatinine criterion, which was defined as an absolute increase in serum creatinine of ≥ 0.3 mg/dL within 48 h or ≥ 1.5 times baseline within 7 days after admission date^21^. Severe AKI was considered as AKI KDIGO stage 2 or 3. The most recent outpatient serum creatinine prior to the admission was regarded as baseline serum creatinine. If outpatient baseline serum creatinine was absent, the Modification of Diet in Renal Disease equation was used to back-estimate baseline serum creatinine level, assuming normal baseline GFR of 75 mL/min/1.73 m^2^, as suggested by the guideline^21^.

### Statistical analysis

Variables were summarized as mean ± standard deviation (SD) for continuous variables and as number with percentage for categorical variables. ANOVA and Chi-squared test was used to compare clinical characteristics and outcomes among admission serum ionized calcium groups, as appropriate. Based on its distribution, admission serum ionized calcium was categorized into six groups; ≤ 4.39, 4.40–4.59, 4.60–4.79, 4.80–4.99, 5.00–5.19, and ≥ 5.20 mg/dL. Restricted cubic spline with 5 knots based on serum ionized calcium was fitted to visualize the possible non-linear relationship between serum ionized calcium at admission and risk of in-hospital AKI. Then, multivariate logistic regression analysis was performed to obtain adjusted odds ratio (OR) of in-hospital AKI occurrence based on admission serum ionized calcium groups, using serum ionized calcium of 5.00–5.20 mg/dL as the reference group because AKI least occurred in the hospital when admission serum ionized calcium was within this range. The adjusting variables were priori-defined and included age, sex, race, baseline eGFR, Charlson Comorbidity Index, comorbidity conditions, admission type, admission service, principal diagnosis, medications, the use for vasopressor and mechanical ventilator at hospital admission, admission serum phosphate, serum magnesium, and serum albumin. P-value was two-tailed with the value of < 0.05 considered statistically significant. All analyses were performed using JMP statistical software (version 14.0, SAS Institute, Cary, NC). The restricted cubic spline was constructed using STATA (version 14.1, StataCorp LLC, Texas, USA).

## Results

### Patient cohort and clinical characteristics

Figure S1 showed the sample selection process of patients included in the study. During the study period, 33,255 patients had available serum ionized calcium measurement at the time of admission. 1,409 ESKD patients, 5,967 patients who had AKI at hospital admission, and 35 patients who had no serum creatinine measurement during hospitalization were excluded. A total of 25,844 patients were studied. The mean age was 61 ± 17 years. Fifty-five percent of enrolled individuals were male. The mean eGFR was 85 ± 23 mL/min/1.73 m^2^. The mean admission serum ionized calcium was 4.8 ± 0.3 mg/dL. The distribution of admission serum ionized calcium was as follows: 8% in serum ionized calcium of ≤ 4.39 mg/dL, 13% in 4.40–4.59 mg/dL, 31% in 4.60–4.79 mg/dL, 29% in 4.80–4.99 mg/dL, 13% in 5.00–5.19 mg/dL, and 6% in ≥ 5.20 mg/dL. Table 1 showed the clinical characteristics of patients based on various admission serum ionized calcium groups.

**Table 1:** Baseline clinical characteristics.

Variables	All	Serum ionized calcium level at hospital admission (mg/dL)						
		≤ 4.39	4.40–4.59	4.60–4.79	4.80–4.99	5.00–5.19	≥ 5.20	p
N	25,844	2017	3,598	8,040	7,457	3,270	1,462	
Age, year	61 ± 17	57 ± 17	61 ± 17	62 ± 17	62 ± 17	61 ± 18	64 ± 17	< 0.001
Male	14,121 (55)	1,038 (51)	2011 (56)	4,567 (57)	4,144 (56)	1692 (52)	669 (46)	< 0.001
Caucasian	23,800 (92)	1813 (90)	3,308 (92)	7,430 (92)	6,887 (92)	3,013 (92)	1,349 (92)	0.01
Charlson score	1.9 ± 2.4	1.8 ± 2.5	1.9 ± 2.5	1.9 ± 2.4	1.8 ± 2.3	1.9 ± 2.3	2.3 ± 2.7	< 0.001
eGFR, ml/min/1.73 m^2^	85 ± 23	90 ± 24	87 ± 24	85 ± 22	85 ± 23	84 ± 24	81 ± 25	< 0.001
**Comorbidities**								
Coronary artery disease Hypertension Diabetes mellitus Congestive heart failure Peripheral vascular disease Stroke	5,220 (20) 13,096 (51) 4,800 (19) 1634 (6) 858 (3) 2031 (8)	279 (14) 860 (43) 294 (15) 94 (5) 33 (2) 98 (5)	599 (17) 1716 (48) 622 (17) 230 (6) 88 (2) 223 (6)	1612 (20) 4,063 (51) 1,424 (18) 565 (7) 280 (3) 599 (7)	1641 (22) 3,901 (52) 1,443 (19) 440 (6) 274 (4) 608 (8)	730 (22) 1734 (53) 694 (21) 199 (6) 131 (4) 341 (10)	359 (25) 822 (56) 323 (22) 106 (7) 52 (4) 162 (11)	< 0.001 < 0.001 < 0.001 0.001 < 0.001 < 0.001
Emergent/urgent admission type	11,352 (44)	1,145 (57)	1,493 (41)	2,821 (35)	3,260 (44)	1793 (55)	840 (57)	< 0.001
Medical admission service	12,970 (50)	1,110 (55)	1581 (44)	3,428 (43)	3,798 (51)	2051 (63)	1,002 (69)	< 0.001
**Principal diagnosis**								
Cardiovascular Hematology/oncology Infectious disease Endocrine/metabolic Respiratory Gastrointestinal Injury and poisoning Other	7,381 (29) 5,445 (21) 671 (3) 720 (3) 1,070 (4) 2,424 (9) 3,679 (14) 4,454 (17)	297 (15) 401 (20) 141 (7) 82 (4) 86 (4) 269 (13) 473 (23) 268 (13)	802 (22) 929 (26) 134 (4) 98 (3) 149 (4) 400 (11) 586 (16) 500 (14)	2,529 (31) 1847 (23) 169 (2) 199 (2) 290 (4) 691 (9) 1,053 (13) 1,262 (16)	2,442 (33) 1,408 (19) 133 (2) 186 (2) 325 (4) 634 (9) 935 (13) 1,394 (19)	933 (29) 574 (18) 56 (2) 80 (2) 146 (4) 306 (9) 473 (14) 702 (21)	378 (26) 286 (20) 38 (3) 75 (5) 74 (5) 124 (8) 159 (11) 328 (22)	< 0.001
**Medication**								
ACEI/ARB Diuretics NSAID	9,378 (36) 9,702 (38) 5,518 (21)	544 (27) 654 (32) 423 (21)	1,220 (34) 1,409 (39) 835 (23)	2,956 (37) 3,220 (40) 1752 (22)	2,851 (38) 2,799 (38) 1571 (21)	1,253 (38) 1,068 (33) 648 (20)	554 (38) 552 (38) 289 (20)	< 0.001 < 0.001 0.01
Vasopressor use	3,444 (13)	322 (16)	520 (14)	1,262 (16)	954 (13)	250 (8)	136 (9)	< 0.001
Mechanical ventilator	6,284 (24)	677 (34)	996 (28)	2,311 (29)	1663 (22)	438 (13)	199 (14)	< 0.001
Serum phosphate^a^, mg/dL	3.6 ± 0.9	3.4 ± 1.2	3.5 ± 0.9	3.6 ± 0.9	3.7 ± 0.8	3.7 ± 0.8	3.6 ± 1.0	< 0.001
Serum magnesium, mg/dL	1.9 ± 0.3	1.7 ± 0.4	1.8 ± 0.3	1.8 ± 0.3	1.9 ± 0.3	1.9 ± 0.3	1.9 ± 0.3	< 0.001
Serum albumin^b^, g/dL	3.4 ± 0.7	3.2 ± 0.8	3.3 ± 0.7	3.4 ± .0.7	3.5 ± 0.7	3.6 ± 0.7	3.4 ± 0.7	< 0.001

Continuous data are presented as mean ± SD; categorical data are presented as count (percentage).

*eGFR* estimated glomerular filtration rate, *ACEI* angiotensin converting enzyme inhibitor, *ARB* angiotensin receptor blocker, *NSAID* non-steroidal anti-inflammatory drug.

^a^Admission serum phosphate was available in 17,062 patients.

^b^Admission serum albumin was available in 6,622 patients.

### Admission serum ionized calcium levels and risk of hospital-acquired acute kidney injury

**Figure 1:**
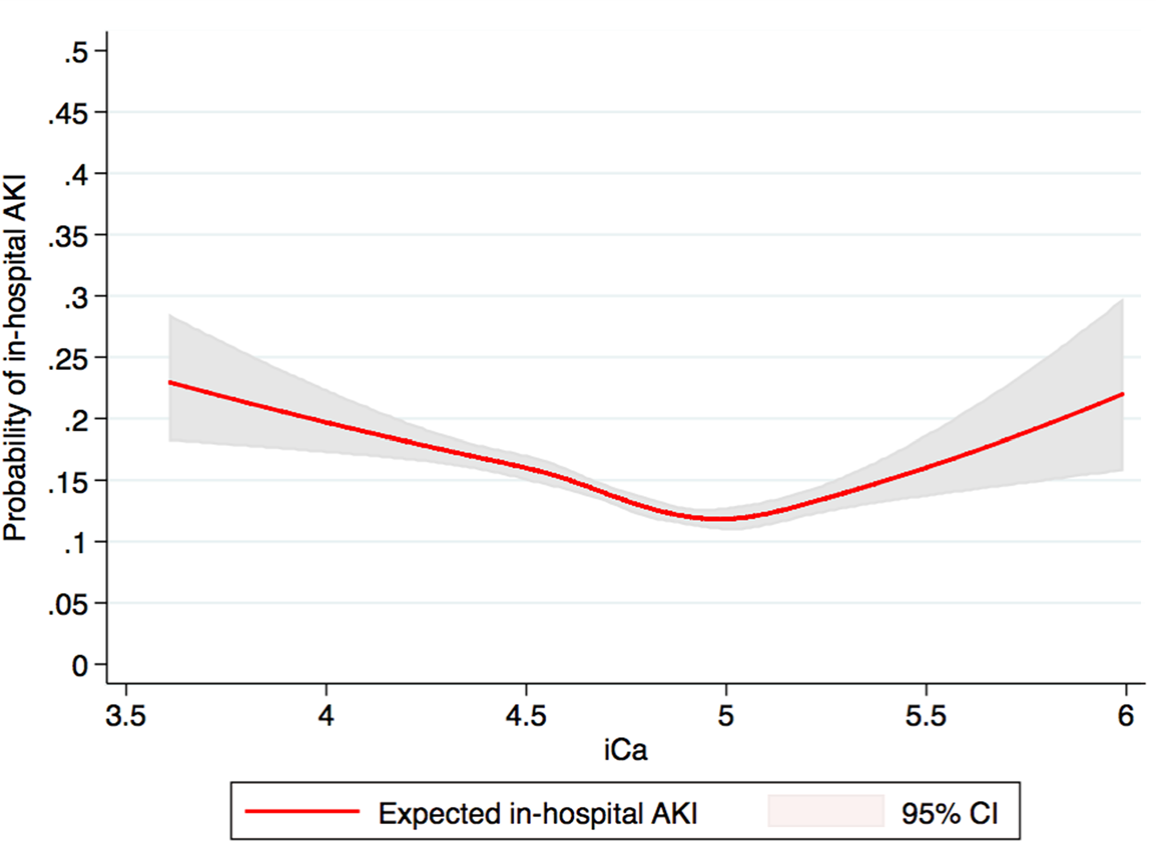
The restricted cubic spline showed U-curved relationship between admission serum ionized calcium and risk of in-hospital acute kidney injury. Figure was created using STATA (StataCorp. 2015. Stata Statistical Software: Release 14. College Station, TX: StataCorp LP).

Of 25,844 patients studied, 3,294 (12.7%) developed AKI in hospital; 10.3% in stage 1, 1.3% in stage 2, and 1.1% in stage 3. 167 (0.6%) patients required renal replacement therapy in the hospital. The incidence of hospital-acquired AKI was 16.5% in patients with admission serum ionized calcium of ≤ 4.39, 14.8% in 4.40–4.59, 12.5% in 4.60–4.79, 11.6% in 4.80–4.99, 10.4% in 5.00–5.19, and 15.0% in ≥ 5.20 mg/dL (Table 2). The restricted cubic spline in Fig. 1 demonstrated a U-curve for the association between admission serum ionized calcium and the risk of hospital-acquired AKI. Both decreased serum ionized calcium of ≤ 4.59 mg/dL and elevated serum ionized calcium of ≥ 5.20 mg/dL were significantly associated with increased risk of hospital-acquired AKI, compared with serum ionized calcium of 5.00–5.19 mg/dL (Table 3a). There was no difference in the risk of hospital-acquired AKI when serum ionized calcium ranged from 4.60–5.19 mg/dL, the normal range of serum ionized calcium. Decreased serum ionized calcium of ≤ 4.79 mg/dL and elevated serum ionized calcium of ≥ 5.20 mg/dL were significantly associated with increased risk of severe hospital-acquired AKI (Table 3b).

**Table 2:** Incidence of in-hospital acute kidney injury based on admission serum ionized calcium levels.

Outcome	All	Serum ionized calcium level at hospital admission (mg/dL)						
		≤ 4.39	4.40–4.59	4.60–4.79	4.80–4.99	5.00–5.19	≥ 5.20	P
AKI	3,294 (12.7)	332 (16.5)	531 (14.8)	1,004 (12.5)	867 (11.6)	341 (10.4)	219 (15.0)	< 0.001
**AKI stage**								
Stage 1 Stage 2 Stage 3	2,672 (10.3) 340 (1.3) 282 (1.1)	227 (11.3) 50 (2.5) 55 (2.7)	423 (11.8) 57 (1.6) 51 (1.4)	819 (10.2) 109 (1.4) 76 (0.9)	726 (9.7) 76 (1.0) 65 (0.9)	301 (9.2) 22 (0.7) 18 (0.6)	176 (12.0) 26 (1.8) 17 (1.1)	< 0.001
Renal replacement therapy	167 (0.6)	36 (1.8)	26 (0.8)	49 (0.6)	35 (0.5)	11 (0.3)	10 (0.7)	< 0.001

**Table 3:** The association between admission serum ionized calcium levels and in-hospital acute kidney injury occurrence.

Admission serum ionized calcium level (mg/dL)	Univariate analysis		Multivariate analysis	
	OR (95% CI)	p	Adjusted OR^a^ (95% CI)	P
**(a) Acute kidney injury**				
≤ 4.39	1.69 (1.44–1.99)	< 0.001	1.45 (1.21–1.74)	< 0.001
4.40–4.59	1.49 (1.29–1.72)	< 0.001	1.33 (1.14–1.56)	< 0.001
4.60–4.79	1.23 (1.08–1.40)	0.002	1.13 (0.98–1.30)	0.09
4.80–4.99	1.13 (0.99–1.29)	0.07	1.07 (0.93–1.23)	0.37
5.00–5.19	1 (reference)	–	1 (reference)	–
≥ 5.20	1.51 (1.26–1.82)	< 0.001	1.26 (1.04–1.54)	0.02
**(b) Acute kidney injury stage 2–3**				
≤ 4.39	4.43 (3.07–6.41)	< 0.001	2.41 (1.63–3.56)	< 0.001
4.40–4.59	2.50 (1.73–3.60)	< 0.001	1.75 (1.20–2.56)	0.004
4.60–4.79	1.90 (1.35–2.68)	< 0.001	1.55 (1.09–2.21)	0.01
4.80–4.99	1.56 (1.09–2.22)	0.01	1.36 (0.95–1.95)	0.10
5.00–5.19	1 (reference)	–	1 (reference)	–
≥ 5.20	2.45 (1.58–3.78)	< 0.001	1.83 (1.17–2.87)	0.008

^a^Adjusted for age, sex, race, Charlson score, baseline glomerular filtration rate, history of coronary artery disease, hypertension, diabetes mellitus, congestive heart failure, peripheral vascular disease, stroke, admission type, admission service, principal diagnosis, use of angiotensin converting enzyme inhibitor/angiotensin receptor blocker, diuretics, non-steroidal anti-inflammatory drug, the need for vasopressor and mechanical ventilator at hospital admission, admission serum phosphate, magnesium, and albumin.

The subgroup analysis based on admission type showed that, among patients with elective hospital admission, only decreased serum ionized calcium ≤ 4.59 mg/dL was significantly associated with increased risk of hospital-acquired AKI, whereas, among patients with urgent/emergent admissions, both decreased serum ionized calcium of ≤ 4.79 mg/dL and elevated serum ionized calcium of ≥ 5.20 mg/dL were significantly associated with increased risk of hospital-acquired AKI (Table S1). However, there was no interaction between serum ionized calcium and admission type on the risk of hospital-acquired AKI (p-interaction = 0.18).

The subgroup analysis based on admission service showed that among patients admitted in medical service, only decreased serum ionized calcium ≤ 4.59 mg/dL was significantly associated with increased risk of hospital-acquired AKI, whereas, among patients admitted in surgical service, both decreased serum ionized calcium of ≤ 4.59 mg/dL and elevated serum ionized calcium of ≥ 5.20 mg/dL were significantly associated with increased risk of hospital-acquired AKI (Table S2). However, there was no interaction between serum ionized calcium and admission service on the risk of in-hospital AKI (p-interaction = 0.73).

## Discussion

The findings of our study showed an independent association between admission level of serum ionized calcium and the risk of in-hospital AKI with a U-curve association. Ionized calcium ≤ 4.59 mg/dL or ≥ 5.20 mg/dL was significantly associated with higher occurrence of in-hospital AKI. For subgroup analysis of the risk of in-hospital severe AKI, our results suggested that ionized calcium ≤ 4.79 mg/dL or ≥ 5.20 mg/dL was significantly associated with higher risk of severe AKI.

Previous studies have demonstrated the AKI risk among patients with various serum calcium levels^14,15,22^. However, total calcium measurements have considerable limitations in the identification of actual calcium abnormalities, such as its dependency on serum albumin level^10^. Our study is the largest cohort to demonstrate the U-curve association between serum ionized calcium and risk of in-hospital AKI. We postulated that abnormality in calcium affects vascular tone, including renal vessels. The imbalance between renal vasoconstriction and vasodilatation, in turn, could result in AKI. Hypercalcemia interferes with the kidneys’ ability to concentrate urine leading to volume depletion with predisposition to AKI^15^. Nonetheless, it could be the degree of severity of the disease that explains this association. Low serum ionized calcium might be related to severity of the illness or sepsis^23–25^. The inflammatory cytokines during the illness impair PTH secretion from parathyroid gland along with resistance to PTH at the end-organs^26^. Furthermore, calcitriol production is also suppressed during this severe illness^27^. These result in low serum ionized calcium. At the same time, severity of the disease is also associated with higher risk of AKI. Among critically ill patients with AKI, recent study demonstrated that low serum ionized calcium levels were independent predictors of all-cause mortality^8^. Further studies are needed to understand more about the mechanistic pathways of this association.

Our study has several limitations. First, the design of the study is observational and retrospective. Hence, causal relationship cannot be established. In addition, some critical information, such as the causes of serum ionized calcium derangements, urine output, and urine electrolytes, were not available or incomplete in our database and, therefore, we were not able to report them. There might be some residual confounders that we unaccounted for when we analyzed the association. Second, we had only single level of serum ionized calcium at the admission so we could not explore whether the time course of changes in serum ionized calcium might influentially affect the risk of AKI. Third, as serum ionized calcium levels at hospital admission were the predictor of interest, only patients with available admission serum ionized calcium were included. However, serum ionized calcium levels were measured in a limited proportion of hospitalized patients at the time of hospital admission (Figure S1). As shown in Table S3, patients with admission serum ionized calcium had lower baseline kidney function, higher comorbidity burden, and were more primarily admitted for cardiovascular and hematology/oncology disease than those without admission serum ionized calcium. Given a potential selection bias, the generalizability of the study finding to patients without admission serum ionized calcium measurement remains unknown. However, strengths of our study should be emphasized. First, we investigated the non-linearity association with restricted cubic spline. Furthermore, we also explored for risk of severe AKI defined as AKI stage 2 and 3 apart from any AKI. Since AKI is associated with increased mortality among hospitalized patients^2,3,5,6^, future studies are needed to assess whether the incorporation of ionized calcium levels in a prognostic model at the time of hospital admission will help identify high-risk patients for AKI during hospitalization. Besides, further trials to investigate the benefit of protocol for correction of abnormal serum ionized calcium levels on the risk of AKI in hospitalized patients may be necessary.

To summarize, admission ionized calcium either ≤ 4.59 mg/dL or ≥ 5.20 mg/dL is significantly associated with a higher risk of in-hospital AKI while admission serum ionized calcium of either ≤ 4.79 mg/dL or ≥ 5.20 mg/dL is significantly associated with higher risk of in-hospital severe AKI.

## Supplementary information

Supplementary Information.
