# Supplementary material for: Impact of admission serum ionized calcium levels on risk of acute kidney injury in hospitalized patients
Source: Sci Rep. 2020 Jul 23;10:12316. doi: 10.1038/s41598-020-69405-0 (PMC7378261; doi:10.1038/s41598-020-69405-0)

Table S1 subgroup analysis based on admission type

1. Elective admission (n=14492)

| Admission serum ionized calcium level (mg/dL) | Univariate analysis | | Multivariate analysis | |
| --- | --- | --- | --- | --- |
|  | OR (95% CI) | p | Adjusted OR* (95 % CI) | P |
| ≤4.39 | 1.49 (1.18-1.89) | 0.001 | 1.33 (1.02-1.72) | 0.03 |
| 4.40-4.59 | 1.40 (1.15-1.70) | 0.001 | 1.33 (1.07-1.64) | 0.01 |
| 4.60-4.79 | 1.06 (0.89-1.27) | 0.49 | 1.03 (0.85-1.24) | 0.78 |
| 4.80-4.99 | 1.04 (0.86-1.25) | 0.66 | 0.99 (0.82-1.21) | 0.95 |
| 5.00-5.19 | 1 (reference) | - | 1 (reference) | - |
| ≥5.20 | 1.35 (1.03-1.76) | 0.03 | 1.05 (0.79-1.41) | 0.72 |

1. Emergent/urgent admission (n=11352)

| Admission serum ionized calcium level (mg/dL) | Univariate analysis | | Multivariate analysis | |
| --- | --- | --- | --- | --- |
|  | OR (95% CI) | p | Adjusted OR* (95 % CI) | P |
| ≤4.39 | 1.91 (1.53-2.40) | <0.001 | 1.59 (1.23-2.07) | 0.001 |
| 4.40-4.59 | 1.48 (1.19-1.85) | 0.001 | 1.29 (1.01-1.65) | 0.04 |
| 4.60-4.79 | 1.37 (1.13-1.67) | 0.002 | 1.29 (1.04-1.59) | 0.02 |
| 4.80-4.99 | 1.18 (0.97-1.43) | 0.11 | 1.15 (0.93-1.42) | 0.19 |
| 5.00-5.19 | 1 (reference) | - | 1 (reference) | - |
| ≥5.20 | 1.70 (1.32-2.18) | <0.001 | 1.51 (1.15-1.97) | 0.003 |

*Adjusted for age, sex, race, Charlson score, baseline glomerular filtration rate, history of coronary artery disease, hypertension, diabetes mellitus, congestive heart failure, peripheral vascular disease, stroke, admission service, principal diagnosis, use of angiotensin converting enzyme inhibitor/angiotensin receptor blocker, diuretics, non-steroidal anti-inflammatory drug, the need for vasopressor and mechanical ventilator at hospital admission, admission serum phosphate, magnesium, and albumin

P-interaction=0.18

Table S2 subgroup analysis based on admission service

1. Medical service (n=12970)

| Admission serum ionized calcium level (mg/dL) | Univariate analysis | | Multivariate analysis | |
| --- | --- | --- | --- | --- |
|  | OR (95% CI) | p | Adjusted OR* (95 % CI) | P |
| ≤4.39 | 1.89 (1.54-2.32) | <0.001 | 1.46 (1.16-1.85) | 0.001 |
| 4.40-4.59 | 1.62 (1.34-1.97) | <0.001 | 1.34 (1.09-1.65) | 0.006 |
| 4.60-4.79 | 1.20 (1.011.43) | 0.03 | 1.07 (0.89-1.29) | 0.47 |
| 4.80-4.99 | 1.11 (0.94-1.32) | 0.22 | 1.08 (0.90-1.29) | 0.41 |
| 5.00-5.19 | 1 (reference) | - | 1 (reference) | - |
| ≥5.20 | 1.41 (1.13-1.77) | 0.003 | 1.22 (0.96-1.55) | 0.11 |

1. Surgical service (n=12874)

| Admission serum ionized calcium level (mg/dL)) | Univariate analysis | | Multivariate analysis | |
| --- | --- | --- | --- | --- |
|  | OR (95% CI) | P | Adjusted OR* (95 % CI) | P |
| ≤4.39 | 1.48 (1.13-1.93) | 0.004 | 1.57 (1.17-2.1) | 0.003 |
| 4.40-4.59 | 1.43 (1.14-1.79) | 0.002 | 1.38 (1.08-1.77) | 0.01 |
| 4.60-4.79 | 1.29 (1.05-1.59) | 0.01 | 1.24 (0.99-1.55) | 0.06 |
| 4.80-4.99 | 1.18 (0.95-1.46) | 0.13 | 1.08 (0.86-1.36) | 0.51 |
| 5.00-5.19 | 1 (reference) | - | 1 (reference) | - |
| ≥5.20 | 1.73 (1.26-2.36) | 0.001 | 1.44 (1.02-2.03) | 0.04 |

*Adjusted for age, sex, race, Charlson score, baseline glomerular filtration rate, history of coronary artery disease, hypertension, diabetes mellitus, congestive heart failure, peripheral vascular disease, stroke, admission type, principal diagnosis, use of angiotensin converting enzyme inhibitor/angiotensin receptor blocker, diuretics, non-steroidal anti-inflammatory drug, the need for vasopressor and mechanical ventilator at hospital admission, admission serum phosphate, magnesium, and albumin

p-interaction=0.73

Table S3 Clinical characteristics between patients with and without admission ionized calcium measurement

|  | No admission ionized calcium measurement | Admission Ionized calcium measurement | p-value |
| --- | --- | --- | --- |
| Age (year) | 61±18 | 62±17 | <0.001 |
| Male | 53% | 55% | <0.001 |
| Caucasian | 93% | 92% | <0.001 |
| GFR (ml/min/1.73m2) | 78±28 | 74±31 | <0.001 |
| Charlson score | 1.8±2.3 | 2.1±2.5 | <0.001 |
| Principal diagnosis   - Cardiovascular - Hematology/Oncology - Infectious disease - Endocrine/metabolic - Respiratory - Gastrointestinal - Injury and poisoning - Other | 21%  15%  3%  3%  4%  9%  15%  29% | 27%  19%  4%  3%  5%  10%  14%  18% | <0.001 |

Figure S1 Study flow chart


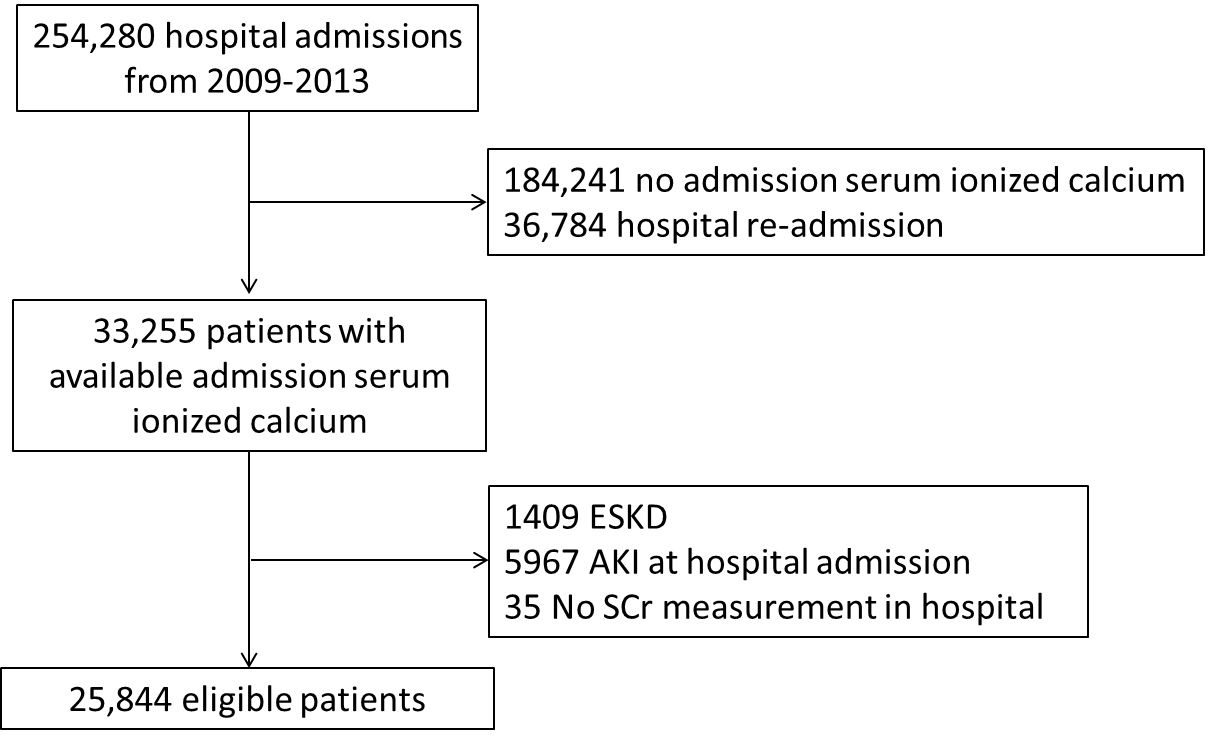

Supplement: Supplementary file 1 — Supplementary Information. [file 41598_2020_69405_MOESM1_ESM.docx]
